# An action-oriented public health framework to reduce financial strain and promote financial wellbeing in high-income countries

**DOI:** 10.1186/s12939-023-01877-8

**Published:** 2023-04-13

**Authors:** Candace I. J. Nykiforuk, Ana Paula Belon, Evelyne de Leeuw, Patrick Harris, Lisa Allen-Scott, Kayla Atkey, Nicole M. Glenn, Elaine Hyshka, Karla Jaques, Krystyna Kongats, Stephanie Montesanti, Laura M. Nieuwendyk, Roman Pabayo, Jane Springett, Aryati Yashadhana

**Affiliations:** 1 Centre for Healthy Communities, School of Public Health, University of Alberta, ECHA 3-300, 11405-87 Ave, Edmonton, AB T6G 1C9 Canada; 2 Centre for Primary Health Care & Equity, University of New South Wales, Level 3, AGSM, UNSW, Sydney, NSW 2052 Australia; 3 Centre for Health Equity, Training, Research & Evaluation (CHETRE), Part of the UNSW Sydney Research Centre for Primary Health Care & Equity, A Unit of Population Health, South Western Sydney Local Health District, NSW Health, Ingham Institute, Liverpool Hospital Locked Bag 7103, NSW, Liverpool, BC 1871 Australia; 4 Provincial Population and Public Health, Alberta Health Services, 2210, 2nd Street SW, Calgary, AB T2S 3C3 Canada

**Keywords:** Financial wellbeing, Financial strain, Social determinants of health, Health equity, Framework, Intervention, Policy, COVID-19 pandemic

## Abstract

**Background:**

Perceived financial security impacts physical, mental, and social health and overall wellbeing at community and population levels. Public health action on this dynamic is even more critical now that the COVID-19 pandemic has exacerbated financial strain and reduced financial wellbeing. Yet, public health literature on this topic is limited. Initiatives targeting financial strain and financial wellbeing and their deterministic effects on equity in health and living conditions are missing. Our research-practice collaborative project addresses this gap in knowledge and intervention through an action-oriented public health framework for initiatives targeting financial strain and wellbeing.

**Methods:**

The Framework was developed using a multi-step methodology that involved review of theoretical and empirical evidence alongside input from a panel of experts from Australia and Canada. In an integrated knowledge translation approach, academics (*n* = 14) and a diverse group of experts from government and non-profit sectors (*n* = 22) were engaged throughout the project via workshops, one-on-one dialogues, and questionnaires.

**Results:**

The validated Framework provides organizations and governments with guidance for the design, implementation, and assessment of diverse financial wellbeing- and financial strain-related initiatives. It presents 17 priority actionable areas (i.e., entry points for action) likely to have long-lasting, positive effects on people’s financial circumstances, contributing to improved financial wellbeing and health. The 17 entry points relate to five domains: *Government (All Levels), Organizational & Political Culture, Socioeconomic & Political Context, Social & Cultural Circumstances, and Life Circumstances*.

**Conclusions:**

The Framework reveals the intersectionality of root causes and consequences of financial strain and poor financial wellbeing, while also reinforcing the need for tailored actions to promote socioeconomic and health equity for all people. The dynamic, systemic interplay of the entry points illustrated in the Framework suggest opportunities for multi-sectoral, collaborative action across government and organizations towards systems change and the prevention of unintended negative impacts of initiatives.

## Background

Commensurate with the strengthening of neoliberal economic policies over time, a growing number of people in high-income countries have struggled to make ends meet and have postponed their financial goals, like homeownership or a graduate degree, to improve their job prospects. Increased living costs, rising inflation, high joblessness, precarization of work, and lack of a living wage have pushed more people into poverty, food insecurity, and housing insecurity or homelessness [1, 2]. Recent austerity measures, such as cuts in social protection programs and provision of basic services, have left people more vulnerable to financial hardship [1–3]. The global economic recession caused by the COVID-19 pandemic aggravated this trend and worsened the gap between the most and least disadvantaged groups [4, 5]; impacts that will be felt for generations.

Individuals and families experiencing cumulative disadvantage due to their social locations (based on intersections of their socioeconomic status, race/ethnic background, gender, sexual orientation, etc.), have been disproportionally affected by the pandemic and related public health measures designed to curtail disease transmission. As a consequence, social and economic recovery will take longer for disadvantaged groups such as Black, Indigenous, and people of color (BIPOC), refugees, young adults, and women [3, 4]. The rising demand among middle-income groups for temporary income and non-cash supports (e.g., food banks) revealed the negative impacts of the pandemic on individual financial circumstances, which have been felt across the socioeconomic spectrum [5]. In the United States, for example, 89.7 million adults reported that paying usual expenses was somewhat or very difficult and 33.7 million Americans were paying their expenses with debt, not income in 2020 [5]. In Canada, recent data showed an increased accumulation of debt, reduction in savings, and growing number of people reporting being unable to cover day-to-day expenses (for shelter, food, and power) or unpredictable expenses (e.g., major home repairs) [6].

The concepts of financial strain [7, 8], financial wellbeing [9–11], and related terms (e.g., financial resilience, financial health) garnered scholarly attention prior to the pandemic, especially in social policy [2, 12] and the financial industry [13, 14]. Yet, these concepts remain ill-defined and vary across disciplines. Consequently, there is no consensus about the measurement and operationalization of financial strain or financial wellbeing [2, 9, 11, 13, 14], rendering research in the area subject to conceptual and methodological inconsistencies. However, financial strain is mostly conceptualized as *subjective* perceptions of one’s current financial circumstances [7, 8]. The experience of financial strain is irrespective of one’s income and assets and may be determined by lifestyle values, life goals, and consumption practices, e.g., a middle-income family feeling financially strained, but not experiencing poverty. In turn, the related concept of financial wellbeing refers to one’s *objective* (actual) *and/or subjective* current and future financial situation [9, 15].

The concepts of financial strain and financial wellbeing are deeply rooted in the consumer finance literature (c.f., Bureau of Consumer Financial Protection (BCFP)) [16]. Mirroring neoliberal governmental agendas and sometimes enveloped in distorted empowerment discourse, financial wellbeing and financial strain programs have been centred to promote *individual-level* financial knowledge, behaviours, skills, and attitudes. This over-emphasis on individual choice and responsibility problematically ignores the structural and systemic barriers that shape access to social and economic rights (e.g., access to healthcare services and education) and obscure governments’ (at all levels) responsibilities for *population-level* public health, economic security, and social vitality.

Often offered by civil society organizations, individual-level programs and services focusing on financial education and counseling tend to have null or short-term goals and outcomes. Because they do not address the systemic, structural determinants of financial circumstances, these behaviour-focused initiatives also may lead to null impacts or, worse, the unintended consequences of perpetuating poverty and income inequity [2]. Public health has a valuable role to play to offset these consequences [13] in four domains. First, financial strain and poor financial wellbeing pose significant threats to physical, social, and mental health and overall wellbeing; for example, they have been associated with suicide mortality [8], heart disease [17, 18], and mental illnesses (e.g., depression) [19, 20] among working age adults. The experiences and impacts of financial strain and financial wellbeing are multidimensional and intersectional. Understanding the mechanisms and the magnitude of the health impact of individual (or family or household) financial circumstances may support the development of initiatives to mitigate financial strain and improve quality of life (e.g., health conditions, life expectancy, happiness, job productivity, and economic prosperity) at individual and societal levels, across the short and longer terms. Second, examining financial strain and financial wellbeing through a social determinants of health (SDoH) lens [21] allows for consideration of the systemic and structural factors shaping people’s abilities to be and feel financially secure – i.e., shifting the focus beyond individual factors to start delineating actions at multiple socioecological levels (e.g., recognizing the interplay and influence of policy and community levels on individuals). Aligned with the SDoH, the commercial determinants of health [22–24] can also contribute to the debate with the close examination of the commercial drivers of financial strain, poor financial wellbeing, social inequities, and physical and mental health issues. A comprehensive view of the harmful health consequences of the global, private sector’s actions can unveil the growing consumption-oriented landscape of the everyday practices and choices orchestrated by corporations [24]. It can also reveal the corporate influence on the public sector [24], which may compromise governments’ ability and willingness to support shared, equitable economic prosperity. Third, incorporating health equity principles into decision-making processes about policy and program design is a critical step in addressing the systematic and structural barriers that contribute to avoidable, unfair differences in health and financial circumstances across the socioeconomic spectrum. Moving away from the one-size-fits-all ‘moment-in-time’ approaches, interventions must be sensitive to the diverse lived experiences, life demands, and challenges faced by all people, with emphasis on groups experiencing disadvantages to better respond to their unique needs over their life course [25]. Fourth, use of the Health in All Policies (HiAP) principles of ‘sustainability’ and ‘synergies between governments and organizations’ can ensure that initiatives related to financial wellbeing and financial strain do not cause harmful health impacts or exacerbate health inequities [26]. When health implications of interventions are systematically considered in policymaking, co-benefits across sectors are optimized with improvement of population health and health equity while society becomes better equipped to respond to health and socioeconomic crises [26].

Despite the critical role of public health in this space, public health research on financial strain and financial wellbeing is very limited [13]. To date, the literature presents some frameworks that offer visual depictions of complex issues [27] related to financial strain and/or financial wellbeing. However, most of these are explanatory frameworks that list determinants [27] of individual behaviour change (e.g., saving, borrowing, spending). In addition, these frameworks are grounded in economic, marketing, and business research as well as the financial industry, missing other sectors implicated in the experiences of financial strain and financial wellbeing. Further, extant frameworks do not adequately account for other social or structural determinants of health, or issues of equity and intersectionality. These frameworks are often simplified representations of influences on an individual’s behaviour, overlooking the interdependent system of cumulative effects and conditions of intersecting social locations. The lack of a population-focused, structural lens diminishes how people’s experiences of social injustice and discrimination may perpetuate and aggravate social and health inequities, further complicating their experiences of financial strain or poor financial wellbeing. Such a public health framework presenting a structural orientation alongside multi-sectoral, high impact areas to effectively reduce financial strain and promote financial wellbeing is currently missing. To address this significant gap in knowledge and action, we developed an Action-Oriented Public Health Framework (herein called Framework) to: reinforce the multidimensional nature of financial wellbeing and financial strain; broaden understanding of the dynamic interconnections between individual financial decisions and their limiting or enabling structural factors; and enhance capacity for organizations and governments to design, implement, and evaluate effective policies, programs, and services to mitigate financial strain and promote financial wellbeing. This paper presents the methodology used to develop the Framework, identifies the financial strain and financial wellbeing definitions that are well-aligned with a SDoH agenda, describes the Framework components, specifies target users, and explains the purpose of, and how to use, the Framework.

## Methods

**Fig. 1:**
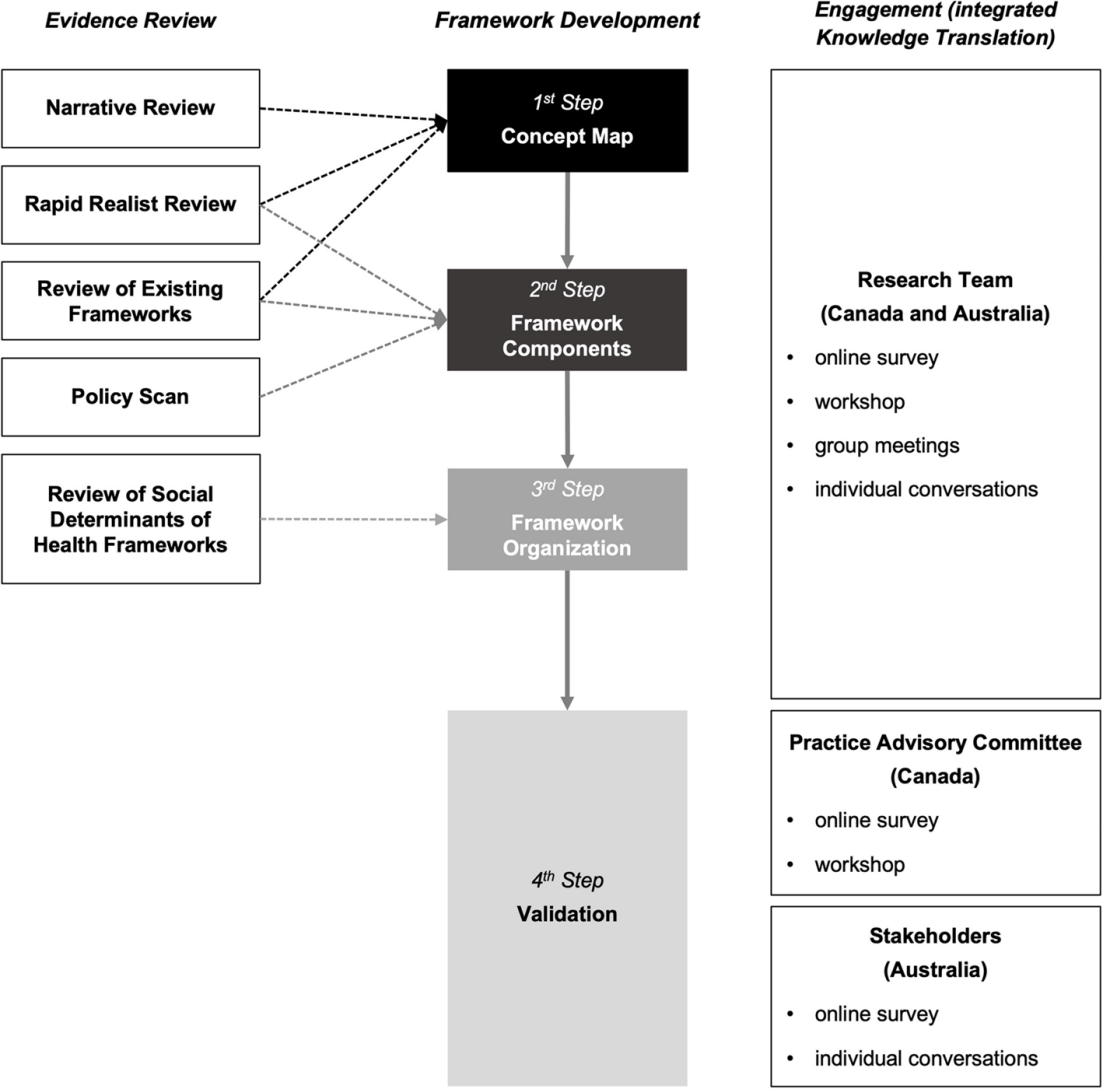
Framework Development and Validation Process

This multi-method research project was conducted by an international team (Canada and Australia) and supported by a national rapid research funding opportunity in response to the COVID-19 pandemic. As such, the evidence-based Framework was developed over an intensive nine-month period in 2020–2021. It involved a 4-step approach (Fig. 1). First, we created a concept map to clarify the definition of financial strain and financial wellbeing, in which the Framework would be grounded. Once we defined this foundation for our work, we moved to the identification of the central pillars of the Framework. In the second step, we reviewed evidence to support the development of the components of the Framework through three research activities: a) examining existing financial strain and financial wellbeing related frameworks available in academic and practice-based (grey) literature; b) conducting a rapid realist review on financial strain and financial wellbeing related initiatives in high-income countries; and c) scanning municipal, state/provincial/territorial, and federal policies on financial strain and financial wellbeing. In the third step, we organized the components of the Framework in light of SDoH and health equity frameworks. The fourth step involved refinement and validation of the Framework with multi-sectoral stakeholders in Canada and Australia. Representatives of government, community, and professional organizations were engaged throughout the research process via online group and individual meetings, online workshops, and/or survey questionnaires. We provide detailed information about these steps below.

### 1^st^ Step: Concept map

Given the lack of consensus on the definitions of financial strain, financial wellbeing, and other related terms (e.g., financial stress, financial health), we created a concept map [28]. This approach allowed for visual representation of key concepts and their interrelationships in a study of complex phenomena. As such, our concept map illustrated the semantically related terms to financial strain and financial wellbeing and the relationships between them according to theoretical and empirical findings in the literature. The main purpose of the concept map was to identify a definition of financial strain and financial wellbeing that would be well aligned with the principles of health equity [25, 29] and SDoH [21]. The concept map also helped us to identify the scope and target areas of the financial strain and financial wellbeing initiatives.

To create the concept map, AB and NG first conducted a narrative review of theoretical papers about financial strain and financial wellbeing and their related terms (e.g., financial stress, financial wellness). Further concepts and definitions were identified through two other simultaneous research data extraction activities: 1) from existing frameworks related to financial wellbeing and financial strain (Step 2a); and 2) from the resources included in the rapid realist review (Step 2b). We also screened the reference lists of included papers in the rapid realist review for additional relevant publications.

### 2^nd^ Step: Development of framework components

Next, we identified the diverse factors that affect financial wellbeing and financial strain to inform the development of the Framework components. We used three concurrent data collection methods: a policy scan; a rapid realist review; and a review of existing frameworks related to financial strain and financial wellbeing. These methods are described below, followed by an explanation of the logic model used to integrate findings into the Framework.

#### Policy scan

KA, KK, AY, NG, and KJ conducted a policy scan to gather contextual information of financial strain and financial wellbeing-related policies at all government levels (i.e., federal, state/provincial/territorial, and municipal) enacted or amended in Australia and Canada since the onset of COVID-19 pandemic. To align with the timelines of the rapid realist review and the overall project, the policy scan was limited to the period of December 2019-December 2020. Publicly available, formal policy documents (e.g., bylaws, strategies, plans) written in English or French were included. In addition to policy databases (e.g., Capital Monitor in Australia and Can-Lii in Canada), we performed targeted web searches of government websites using Google Advanced engines and manual searches.

The strategy used to select municipal or local governments for inclusion in the scan was tailored to each country. In Canada, we included 31 municipalities systematically selected for the Canadian Against Cancer’s Prevention Policies Directory (PPD) – an existing evidence-based policy tracker with the goal of achieving a pan-Canadian range of jurisdictions [30]. To supplement that source, we consulted the Big City Mayor’s Caucus (which lists representatives from the 22 biggest Canadian municipalities) and three new municipal members of the Urban Public Health Network not already included in the PPD. The final sample included 39 jurisdictions, representing a diverse subset of Canadian municipalities. In Australia, we first selected the largest Major Urban Centre Local Government Areas (LGA) of each of the eight states and territories. The remaining LGAs were sorted out according to two indexes: (1) Remoteness Area Rating classification (Major Cities, Regional, Remote); and (2) Socio-Economic Indexes for Areas (SEIFA) decile score (where 1 is equivalent to most disadvantage). We used a cluster-randomised sampling technique to ensure the geographic and socioeconomic representativeness of LGAs selected. That process resulted in the selection of 40 Australian LGAs.

After primary and secondary screening, descriptive data (e.g., jurisdiction, target population) and classification of policies according to type of intervention (universal, targeted, or proportionate) were recorded in an Excel spreadsheet as part of data extraction. In total, we included 213 policy documents in Canada and 97 in Australia. KK created Evidence and Gap Maps (EGM) [31] to organize federal, state/provincial/ territorial, and municipal policies according to the target areas (e.g., food/nutrition, housing, caregiving, and transportation) and target populations (i.e., general population, equity-seeking populations, and privileged groups). These maps allowed for identifying common areas of intervention and gaps in the political response to the pandemic. Research team members discussed EGM and summary of findings.

#### Rapid realist review

NG, AY, and KJ performed a rapid realist review (RRR) [32, 33] of the academic and practice-based (grey) literature of initiatives related to financial strain and financial wellbeing conducted in developed countries [34] between 2015–2020. We followed Preferred Reporting Items for Systematic Reviews and Meta-Analyses (PRISMA) [35] and Realist and MEta-narrative Evidence Syntheses: Evolving Standards (RAMESES) [36] guidelines. Peer-reviewed papers were searched in: MEDLINE, PsycINFO, and Web of Science (Social Science Citation Index). We used ProQuest, Informit, and Google Advanced to search practice-based papers. NG and AB independently screened the titles and abstracts of the academic literature for inclusion (*n* = 3516). AY and KJ did the same for the practice-based sources (*n* = 6035). Full-texts of potentially relevant papers were screened by a reviewer (NG for academic literature; AY for practice-based literature). A 10% sample was reviewed by another independent reviewer (AB for academic literature; KJ for practice-based literature). Conflicts were resolved through discussion until reaching consensus and involved a third reviewer from the research team when needed.

For data extraction and synthesis of selected peer-reviewed papers (*n* = 39) and practice-based papers (*n* = 36), we used EPPI-Reviewer software. We appraised the peer-reviewed sources with the Mixed Methods Appraisal Tool (MMAT) [37]. An in-depth abductive analysis [38] was done in NVIVO 12 [39]. This critical realist analysis allowed for identification of the underlying causes of a social phenomenon, known as generative mechanisms [40] guided by an existing realist health equity framework [41]. In the sources screened and/or reviewed in the RRR, we identified further materials listed in the reference lists that were deemed relevant to the Framework development. We added these sources to a separate list for review; when appropriate and relevant, we used in the preparation of concept map (Step 1). In the analysis of RRR data, AY, NG, and KJ considered the underlying program theories of the interventions (units of analysis) and examined the evidence to better understand on what works, for whom, and under what contexts/conditions. A report was written summarizing the context, mechanisms, and outcomes (CMO) relationships behind the initiatives included in the RRR (i.e., neoliberal ideology, social location and difference, and social equity discourse). Research team members then supported further interpretation of findings. The analysis of the CMO relationships is published elsewhere (forthcoming).

#### Review of existing frameworks related to financial strain and financial wellbeing

AB conducted a review of the academic and practice-based literature on financial strain and financial wellbeing related frameworks. The research team was invited to suggest additional frameworks. In total, we included 14 explanatory, interactive, and/or action-oriented frameworks [27] from different areas of knowledge and practice (e.g., social policy; anthropology; and business, commerce, and marketing). A few frameworks were authored or commissioned by institutions (see, for example, Financial Consumer Agency of Canada [42] and Indigenous Consumer Assistance Network Ltd. [43]).

AB developed a form to capture and assess critical elements of each framework, including their strengths and limitations. The assessment was done through the lenses of SDoH, health equity, and HiAP. To complement this approach, AB undertook a careful examination of other determinants discussed in the texts, but not depicted in the visual representations of the frameworks; she then developed visual maps of the relationships between the elements. A summary of data analysis of the frameworks was then shared with the research team for further discussion of their strengths and limitations.

#### Evaluation logic model

For coordinated integration purposes, AB led the drafting of the Framework with the research team providing feedback throughout its development. Applying a realist synthesis approach (see de Leeuw et al., 2015 [44] for an example) and informed by principles of health equity [25, 29] and HiAP [26], we developed an evaluation logic model to identify priority areas for action on financial strain and financial wellbeing. First, from the RRR portion, the generation of context-mechanism-outcome configurations led to the identification of 80 emergent themes related to interventions on financial strain and financial wellbeing. Building upon the frameworks reviewed in Step 2c, AB grouped themes that were heuristically related to one another, which were then used to map and define key actionable areas. Using the EGM created in the policy scan, AB identified the areas that were not present or underrepresented in the RRR findings*.* The research members who conducted the RRR and policy scan carefully reviewed the groupings. The other research team members guided this process and provided critical input for the groupings. Together, we developed the Framework components, herein called *entry points for action*.

### 3^rd^ Step: Organization of the framework

We purposively selected frameworks on SDoH and health equity (see, for example, Public Health Agency of Canada’s tool [25]) to support the organization of the components and to ensure our preliminary model was tailored into an Action-Oriented Public Health Framework that is aligned with public health agenda and practice. As we organized the Framework, we closely examined the distal and proximal influencers (e.g., age and ageism; occupation and labour market) that are known to be related to material circumstances and socioeconomic status. Given the absence of financial strain and financial wellbeing in the SDoH and health equity frameworks, our assumption was that broader systemic and structural influences on material circumstances and socioeconomic status hold analogous relationships with financial strain and financial wellbeing. Likewise, the pathways between material circumstances and socioeconomic status and ill-health were used to contribute to our understanding of the unique interrelationships between financial strain and financial wellbeing and other social determinants, human health (physical, mental, and social), and overall wellbeing. Using these considerations, we grouped the entry points for action into *domains*.

### 4^th^ Step: Validation

We adopted an integrated Knowledge Translation (iKT) approach in the development, refinement, and validation of our Framework. In addition to the ongoing input from the 14 research team members (two principal investigators, six co-investigators, and six highly qualified research staff in Canada and Australia), we gathered feedback on the draft Framework from 16 Practice Advisory Committee (PAC) members in Canada and six stakeholders in Australia (Fig. 1). These content and implementation experts represented diverse areas of knowledge and practice expertise from community and professional organizations and government sectors (e.g., healthcare services, justice system, financial institutions). We gathered their feedback at multiple stages and through various modes. We incorporated their input into the Framework to ensure that it is user-friendly and responsive to the needs of multiple target users in different contexts.

We held two separate virtual workshops (each two-hours long) via Zoom: one with the research team members (CDA and AUS) and another one with the PAC members in Canada. In preparation for the workshops, we sent out a package with an executive summary of the Framework and an online questionnaire with three open-ended questions about relevance, utilization, and comprehensiveness (e.g., improvements needed, including unclear or missing aspects). Those questions were asked again at the workshops with the research team and PAC members. CN led the workshops. To facilitate a more engaging discussion, the group was divided into three break-out rooms. AB, KK, and NG facilitated and audio-recorded the break-out sessions. At the end of the break-out sessions, all attendees were sent back to the main room to present their main discussion points. We used Miro software [45] to capture and share the collective reflections on the Framework with all members at the large group discussion so all attendees could learn and react to different perspectives. Examples of topics discussed were the need to include power dynamics; emphasize the opportunities for partnerships; and, clarify the relationships between the components depicted in the Framework. Due to low participation rate at recruitment (as a result of pandemic-related pressures experienced by stakeholders), instead of the virtual workshops, the Australian stakeholders participated in a virtual one-on-one meeting and/or a telephone conversation with either AY or KJ who briefly described the Framework. The stakeholders then received the executive summary and a link to a modified version of the survey questionnaire to provide feedback. We created a single file to combine the individual feedback from the research team, PAC members, and Australian stakeholders along with the collective feedback recorded in Miro software in the workshops. The analysis informed the further development and refinement of our Framework.

The University of Alberta Research Ethics Board (Pro00102631) and the University of New South Wales Human Research Ethics Committee (HC200896) approved this project. All participants received a project information letter and consent form prior to the workshops and individual conversations. At the onset of such activities, CN reviewed the documents with the participants with time for questions about the project. All participants provided informed consent, which was implied by the overt action of completing the survey and participating in the workshops or individual conversations. This process was cleared by both research ethics boards.

## Results

### Definitions of financial strain and financial wellbeing

To lay the foundation of our Action-Oriented Public Health Framework, we adopted Salignac et al.’s definition of financial wellbeing: “when a person is able to meet expenses and has some money left over, is in control of their finances, and feels financially secure, now and in the future” (p.1596) [15]. We selected this definition for three reasons. First, it is informed by an ecological life-course perspective and, therefore, is better aligned with public health values of health equity and SDoH principles. It considers: a) the interrelationships between factors at individual and structural levels; and b) the trajectories during an individual’s lifetime shaped by expected or unexpected events (e.g., birth of a baby, serious occupational injury). Second, this definition combines objective and subjective measures: e.g., income and savings for regular or unexpected expenses and discretionary spending; and perceived control over finances and financial security. Third, it is inclusive of perceptions regarding present and future financial conditions (e.g., day-to-day money management and planning of financial future), which is consistent with the financial wellbeing literature [9, 10] and practice (see, for example, BCFP [16] and Prosper Canada [46]).

We used the definition of financial strain as the perception of being unable to cope financially given their current financial circumstances, which may cause anxiety or worry [8]. In this regard, financial strain is part – not the antonym – of financial wellbeing. Reducing financial strain is one critical strategy towards improving financial wellbeing. A synonym of financial stress or financial distress, financial strain is about feelings and perceptions; and, therefore, can be unrelated to objective measures of wealth, such as income levels and ownership of assets. For instance, a young adult objectively classified as living below the poverty threshold may not feel under financial strain whereas a middle-income family with children may feel financially strained because of mortgage debt and student loans.

### The action-oriented public health framework to reduce financial strain and promote financial wellbeing

#### Framework components

**Fig. 2:**
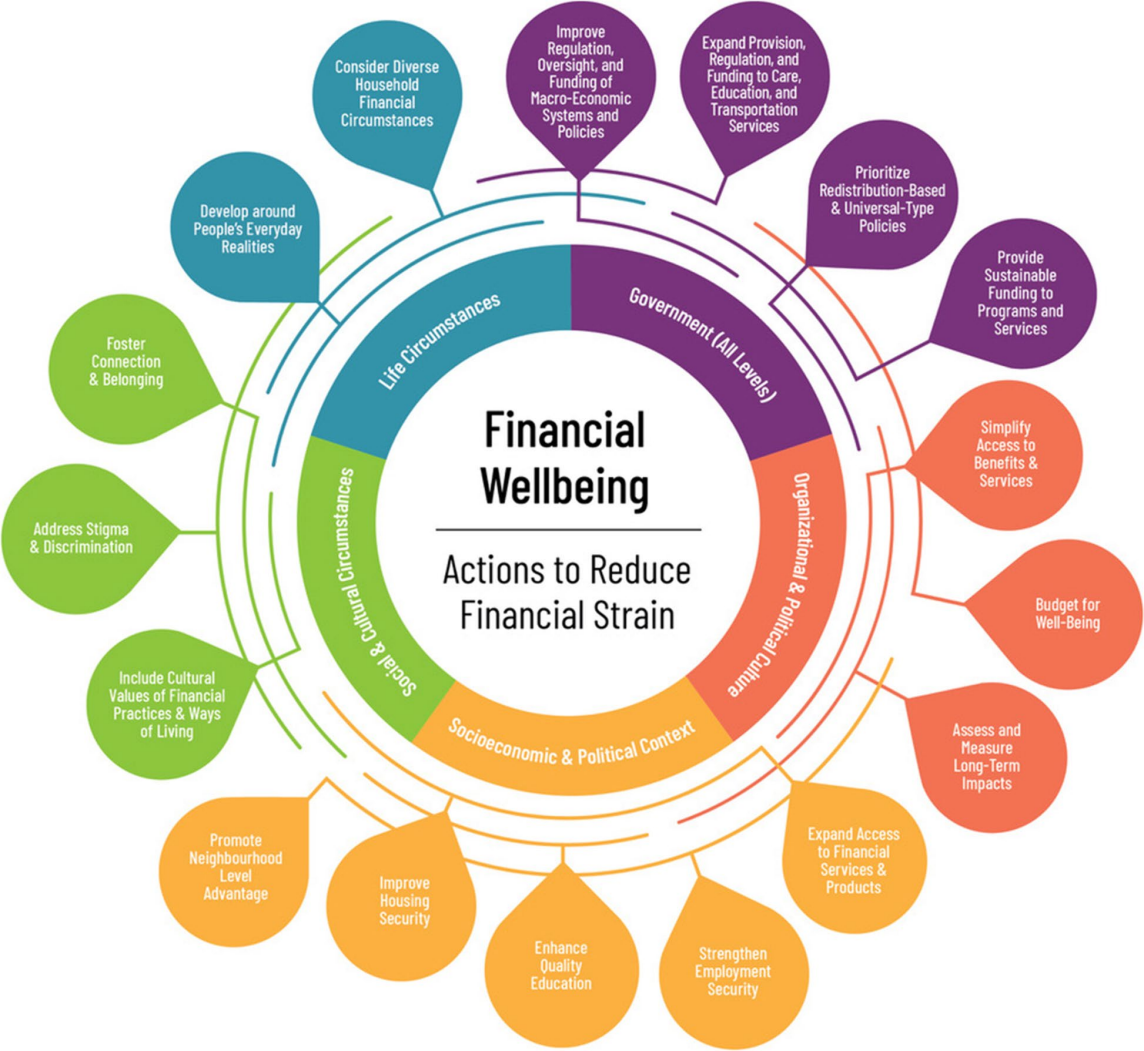
Action-Oriented Public Health Framework on Financial Wellbeing and Financial Strain

We positioned financial wellbeing and reduction of financial strain at the heart of the Framework, representing both the goal and outcome of the initiatives implicated by each entry point for action (Fig. 2). We identified five interrelated domains suggesting where to act: *Government (All Levels)*, *Organizational and Political Culture*, *Socioeconomic & Political Context*, *Social & Cultural Circumstances*, and *Life Circumstances*. The Government (All Levels) domain is meant only for the public sector, including municipal, state/provincial/territorial, and federal governments. The other four domains present actions that can be undertaken by either governments or organizations. In total, there are 17 different, but interconnected entry points for action linked to the domains. The entry points indicate what to do in each domain. For instance, organizations and governments designing, delivering, and/or assessing (evaluating) initiatives related to Organizational and Political Culture domain should seek to ‘Simplify Access to Benefits & Services’, ‘Budget for Wellbeing’, and ‘Assess and Measure Long-Term Impacts’. Table 1 outlines definitions of the domains and entry points for action.

**Table 1:** Domains and entry points for action and their definitions in the action-oriented public health framework on financial wellbeing and financial strain

DOMAINS & ENTRY POINTS FOR ACTION	DEFINITIONS
***Government (All Levels)***	*This domain targets structural actions that can be taken by governments through the governing systems of public and private sectors. It refers to macroeconomic, public, and social policies as well as underlying power structures*
Improve Regulation, Oversight, and Funding of Macro-Economic Systems and Policies	Increase effectiveness and impact of government regulation and oversight of financial sector, housing market, and employment and labour market. Ensure adequacy of funds for sustainable actions
Expand Provision, Regulation, and Funding to Care, Education, and Transportation Services	Ensure governments oversee, regulate, and provide guaranteed level of adequate funding to quality essential care (e.g., childcare, health care), education, and transportation services
Prioritize Redistribution-Based & Universal-Type Policies	Build and improve equity-based policies that redistribute wealth or span the socioeconomic spectrum. Such policies (e.g., progressive taxation, universal basic income, and raising minimum retirement pension) disproportionately benefit people who experience disadvantage
Provide Sustainable Funding to Programs and Services	Ensure continued and appropriate amount of public financial assistance to support operations and service delivery infrastructure of organizations and governments, targeting areas that directly or indirectly impact people’s financial circumstances
***Organizational & Political Culture***	*This domain targets those processes that affect the delivery and sustainability of government, organizational, and community actions. It involves consideration of organizational culture and power dynamics*
Simplify Access to Benefits & Services	Remove barriers and bureaucratic ‘red tape’ that limit people’s access to benefits, programs, and services, including communication barriers (e.g., low literacy levels), strict contingencies (e.g., work-for-welfare), restrictive eligibility criteria, and onerous assessments (e.g., to qualify for disability benefits)
Budget for Wellbeing	Create budgets that prioritize long-term human wellbeing over financial outcomes alone (e.g., balancing budgets through austerity measures that negatively impact health and overall wellbeing)
Assess and Measure Long-Term Impacts	Use measures of human wellbeing to understand the long-term impacts of policies, programs, and services (e.g., social impact) Take a long-term approach to evaluation (e.g., cost–benefit analysis)
***Socioeconomic & Political Context***	*This domain targets social and political actions. It encompasses changes to the political and community landscape that, together, shape the availability of resources, opportunities for poverty reduction, possibilities for growth of the middle-class, and improvements in the distribution of power at the societal level*
Expand Access to Financial Services & Products	Increase access to mainstream and alternative financial services and products that are inclusive, culturally appropriate, affordable (e.g., low-fee or no-fee), flexible in terms of contracts and transactions, and responsive to people’s needs and circumstances Facilitate access to information about mainstream and alternative financial services and products
Strengthen Employment Security (Income and Benefits)	Improve access to stable, well-paid, and regulated jobs with employee benefits programs for all workers
Enhance Quality Education	Facilitate access to education and training to improve people’s long-term income prospects
Improve Housing Security	Strengthen affordable housing policies, including high quality options for public housing Increase access to diverse affordable and supportive housing options in order to provide people with dignified choices that fit their needs
Promote Neighbourhood-Level Advantage	Increase neighbourhood-level access and opportunities for education, employment, safety, and security (e.g., addressing high exposure to the criminal justice system or providing meaningful supports for poorly funded public amenities) Target family, community, and neighbourhood through multi-level initiatives to improve local services and supports
***Social & Cultural Circumstances***	*This domain is about political, community, organizational, and individual actions that shape or recognize social and cultural contexts, hierarchies of power, and people’s social backgrounds and identities (e.g., immigration status, gender, sexual orientation, race/ethnicity) that accumulate to impact their financial circumstances*
Include Cultural Values of Financial Practices & Ways of Living	Recognize and respect the complexity and diversity of cultural values attributed to financial resources (e.g., money, goods) and financial transactions Build initiatives that recognize the symbolic and economic values of different ways of being and doing (e.g., pay for informal caregiving)
Address Stigma & Discrimination (e.g., systemic racism and ableism)	Build initiatives to explicitly reduce stigma and discrimination of groups who experience cumulative disadvantage across the lifespan (e.g., racialized people) and intersecting challenges (e.g., Indigenous woman experiencing disability) in financial services, job markets, and school or workplaces Address financial abuse and barriers to both financial independence and intergenerational wealth-building that disadvantaged groups have systematically experienced
Foster Connection & Belonging	Enhance community capacity, empowerment, and connections through community-led or participatory approaches promoting social capital and social cohesion
***Life Circumstances***	*This domain targets political, community, organizational, and individual actions that impact people’s complex life circumstances, multiple roles, and power relationships (e.g., individual agency and power within a household) that come together – positively or negatively – to shape their financial situation*
Develop around People’s Everyday Realities	Remove barriers to enrolment and participation in financial strain and financial wellbeing related initiatives (e.g., access to childcare, transportation costs) Ensure the timing and content of the initiatives are tailored to the target populations. Consider people’s values, life stages, life demands, and daily roles and responsibilities
Consider Diverse Household Financial Circumstances	Create initiatives that are appropriate to people’s current financial circumstances, particularly for people experiencing poverty and facing unmet basic needs (e.g., food insecurity, energy insecurity, housing insecurity) Set realistic, achievable goals (e.g., building savings only after basic needs are addressed)

We used coloured lines to represent the interrelationships between the five domains. The lines cross different domains to suggest an action in one domain may have expected or unexpected effects on other domains. For instance, an action in one domain may lead to unintended negative or positive effects on another, which may influence levels of financial strain and financial wellbeing. The coloured lines enhance the end user’s understanding of the need for cooperation and collaborative work and is meant to encourage partnerships across sectors to accomplish shared goals – and population impacts – together.

#### Aims

The evidence-based Framework aims to:

- Advance knowledge on the complex, dynamic interconnectedness of political, socioeconomic, and cultural determinants of financial wellbeing and financial strain at the individual and population levels.
- Introduce key domains and entry points for action that are more likely to lead to long-term positive impacts on people’s financial situations.
- Help end users select high-impact actionable areas for individual or collective action aligned with the mandates and scope of practice of organizations and government sectors.
- When co-applied with a system-thinking approach, contribute to the identification of obstacles, weaknesses, and unintended harms of chosen initiatives.
- Support embedment of equity and intersectionality considerations across governmental or organizational decision-making processes to minimize unintended negative consequences and maximize positive effects on financial wellbeing of different population groups, particularly those experiencing disadvantage.
- Assist with situating the financial wellbeing or financial strain initiatives within the broader context of multiple existing polices, programs, and services to better reveal how they relate to one another in a whole-of-society approach.
- Encourage forging and strengthening of intersectoral collaboration through transformative partnerships for coordinated, effective, and sustainable response to poor financial wellbeing and financial strain.

#### Utilization

The Framework is not meant to be prescriptive and can be used flexibly across contexts, including diverse organizational mandates and portfolio boundaries. We encourage organizations and governments to use the Framework as a tool to reflect on and discuss ongoing or planned initiatives for targeted or integrated action on financial wellbeing and financial strain. The Framework may support decision-making at any stage of the initiative (i.e., design, implementation, and assessment or evaluation) and for different purposes (i.e., intervention, policymaking, advocacy, or research). Regardless of level of the initiatives (upstream, midstream, or downstream), their focus (e.g., financial literacy, housing security, gender equity), and their target audiences (e.g., seniors, low-wage workers, Indigenous women), the Framework may help end users identify what needs to be done and who may need to be involved to strengthen their intended actions and produce long-lasting positive outcomes.

Given that it is neither feasible – nor sometimes desirable – to act on all domains and entry points simultaneously, we encourage organizations and government sectors to identify which domain(s) is best aligned with their scope of practice and core mandates, and to utilize the strategies therein according to their needs. A suggested step-by-step process on how to use the Framework is presented elsewhere [47]. To support effective use of the Framework, we outline some considerations and suggest illustrative prompts for each stage of an initiative (Table 2).

**Table 2:** Guide for utilization of the action-oriented public health framework on financial wellbeing and financial strain

STAGE OF THE INITIATIVES	STEPS	PROMPTS
1. Design	If at the design stage of an initiative, identify domain(s) and respective entry point(s) for action that are aligned with the core mandate and scope of practice of the organization or government sector Skip to **Stage 3**	• What are the priority areas for the organization or government sector? • What actions can the organization or government sector take?
2. Re-Design	If the initiative is underway, compare its scope and activities with the definition(s) of the entry point(s) of action selected Then, reflect on potential gaps in the initiative’s current approach, including the need to strengthen actions and/ or address potential unintended consequences that may have arisen	• What are any gaps or weaknesses? • What is the nature of any (potential) unintended negative impacts?
3. Implementation	Consider the capacity of the organization or government sector to act on the area(s) selected in **Stage 1** or to make appropriate changes in the ongoing actions as identified in **Stage 2**	• To what extent does the organization or government sector have resources to act on the area selected? • For ongoing initiatives, does the organization or government sector have resources if further action or changes in the initiative are deemed necessary? If not, go to **Stage 4**
4. Addressing Gaps and Forming Partnerships	If relevant, consider forging and/ or strengthening partnerships with other organization(s) or government sector(s) who share the same goals to aim for coordinated action	• Who needs to be at the decision-making table? Consider: • going outside practice/discipline-specific boundaries • using common language for effective communication • incorporating lived experience perspectives of target groups
5. Expanding Action and Forming Partnerships	If relevant and feasible, identify other domain(s) and corresponding entry point(s) for action that can be integrated into the initiative Then, consider opportunities for collaboration across departments, organizations, and/or communities to promote efficient, effective, and sustainable changes	• What else that should be done to ensure goals are successfully achieved? • Who are the other potentially relevant actors? How can they be meaningfully engaged?

#### Target users

Our comprehensive, yet visually simple Framework was developed for private, community, civil society, and non-for-profit organizations (e.g., public health agencies, advocacy services, trade unions) as well as municipal, provincial/territorial/state, and federal governments working on areas directly or indirectly linked to financial strain and financial wellbeing (e.g., child and family supports, financial inclusion programs, reemployment services). It is applicable to a wide range of downstream, midstream, and upstream initiatives. For acknowledging multiple ways of being and doing across diverse everyday realities, this Framework is more inclusive and, therefore, more responsive to the needs of initiatives targeting different audiences (e.g., Two-Spirit, Lesbian, Gay, Bisexual, Transgender, Queer, Intersex and Asexual communities (2SLGBTQI2A+), Black communities, youth) than other frameworks on financial strain. The Framework can also be used for research purposes to support exploratory analysis of the determinants of financial wellbeing and financial strain and their impacts on people’s living conditions, health, and overall wellbeing.

## Discussion

In this multi-method project, we actively collaborated with research team members and representatives of governments and organizations to develop a theoretically-informed and empirically derived action-oriented public health Framework to inform policies, programs, and services aiming to address the causes and consequences of poor financial wellbeing and financial strain. The COVID-19 pandemic has brought greater societal recognition of the broad systemic and structural influences on financial wellbeing – e.g., gig economy, shortage of affordable housing and high costs of quality childcare – that impose barriers to people’s wealth building and financial security. While common first responses to the pandemic-related economic recession were to alleviate the imminent financial hurdles [48], building back fairer [49] will require actions that target the root causes of financial strain and poor financial wellbeing. Initiatives should move beyond the rationale of borrowing, spending, saving model, in which individuals are to learn how to make *rational financial choices*. Sociocultural and structural factors may influence individual financial behaviours, knowledge, and decisions on what, where, and how to use their financial resources; however, people may not enjoy the ultimate freedom to make those financial decisions [12]. Additionality, interventions focusing on behavioural change place the onus of finding *solutions* on the individuals experiencing disadvantage, diverting attention away from governments’ responsibilities to address structural inequity and exclusion. Elevated levels of financial strain and poor financial wellbeing created before, but aggravated by the pandemic, as well as those initiated during the pandemic (e.g., through loss of livelihood), will have an adverse generational impact that requires a corollary, sustained social and public health response.

Situated in the public health realm, the Framework development was informed by principles of health equity [25] and HiAP [26] and was grounded in SDoH program logic [21]. Our Action-Oriented Public Health Framework presents 17 high-impact areas (i.e., entry points for action), organized into five domains, to support government and organization actions directly or indirectly related to financial wellbeing and financial strain. It integrates financial wellbeing and financial strain within a broader context of governmental agendas, organizational culture, socioeconomic and political factors, sociocultural values, and life circumstances. It moves beyond the individual-level interventions on financial education and financial inclusion to incorporate structural factors enabling or limiting people’s opportunities and capabilities to enjoy financial wellbeing. It presents multiple entry points through which diverse organizations and government sectors can align actions on financial wellbeing and financial strain within their respective mandates and scopes of practice. This can prompt new possibilities for collaboration and intersectoral action on these cross-cutting issues. Equitable response is central to the Framework; therefore, in addition to informing actions to ameliorate financial wellbeing and reduce financial strain across the socioeconomic spectrum, the holistic Framework supports initiatives targeting groups experiencing cumulative disadvantage, such as single mothers and young workers.

To our best knowledge, this is the first public health action-oriented Framework on financial wellbeing and financial strain. Public health researchers, decision-makers, and practitioners have until now been absent from discussions of financial wellbeing as a determinant of health that goes beyond possession of assets and income levels [13]. With this Framework, we bridge the gap between the values of public health initiatives and current programs and services focused on financial management advice and education. Public health can start to more systematically explore the impacts of how people perceive and experience their present and future financial circumstances in addition to and/or in combination with the objective measures of wealth or deprivation. This is essential for preventive action concerning the SDoH interrelated with financial strain, financial wellbeing, and their respective impacts on health and quality of life across the life course, and equitably across different populations living in diverse contexts and circumstances. Such a systematic exploration will reveal deeper, more nuanced knowledge on the relationship between SDoH and people’s financial situation and decisions revealing the dialectical tension between individual agency and external, structural constraints. For instance, this understanding will support considerations that (1) income and assets may not correspond to the adopted ways of life (e.g., a middle-income single adult with no savings or retirement plan) and (2) the amount of financial resources one has does not strictly depend on the wage and assets (e.g., individuals accessing cash loans or borrowing from families, friends, and formal and informal financial services) – and recognizing that information on cash availability or intergenerational wealth are rarely included in public health studies and survey questionnaires. In turn, other sectors, like the financial industry and social policy, can reflect and act on the intertwined relationships between individual and structural factors in the broader, complex contexts shaping financial wellbeing and financial strain. This can be an opportunity to redesign their individual-level initiatives – via, for instance, resource allocation and partnerships – to also tackle more effective areas that will impact populations, for example, the rising costs of higher education. Taken together, all of this can lead to the design, implementation, and assessment/evaluation of more sustainable initiatives generating long-term lasting effects on people’s financial situation.

## Limitations and strengths

Given the rapid nature of this project and the peculiar working circumstances imposed by the pandemic, this research-practice collaboration presents many strengths, but also some limitations. While we were not able to perform a systematic literature review on financial wellbeing and financial strain, we instead combined a RRR and in-depth realist analysis [40] to reveal what works for whom under what circumstances for a better understanding of the social complexities around financial wellbeing and financial strain interventions. Additionally, we convened an intersectoral group across Canada and Australia representing diverse expertise and practice for critical feedback on the draft Framework. Their input supported revisions to the final version of the Framework, ensuring its relevance for practice and policy action. However, we recognize that, while diverse, the representation of the government sectors and organizations who agreed to participate was limited due to the resource and capacity constraints faced by stakeholders at that point of the pandemic. Providing different channels for participation with longer, flexible timeframes may encourage and facilitate engagement of groups underrepresented in this project (e.g., organizations assisting refugees). Different types of incentives (e.g., rewards of monetary values or public recognition) should be explored in future work to ensure that organizations facing staff and resource shortages can take part of such deliberative, participatory processes. Another limitation is that we used academic and practice evidence from high-income countries to develop this Framework. While the resource may be useful for low- and middle-income economies, it may require tailoring of the entry points and domains to reflect particular contexts and address local needs.

The combination of multiple, iterative methods including iKT to yield an evidence-informed Framework was one of the main strengths of this rapid project. Despite the limited amount of time available for this project, we were able to gather and critically assess the latest evidence on initiatives and policies related to financial wellbeing and financial strain and engage with multiple organizations and government agencies to co-develop a much-needed public health Framework informing action on financial wellbeing and financial strain. Additionally, our international research team brought diverse disciplinary and implementation expertise to the development, refinement, and validation of the Framework. The resulting unique, holistic action-oriented Framework addresses a gap in knowledge and action in public health and indicates opportunities for synergies and cooperation across sectors, agencies, and communities sharing the same goals. It successfully integrates SDoH and health equity lenses as well as HiAP principles into a practice and policy field that was until now confined within financial industry and social policy.

## Conclusions

The Action-Oriented Public Health Framework to Reduce Financial Strain and Promote Financial Wellbeing represents our collective efforts to develop a unique framework with diverse potential target users in high-income countries. The Framework is concise and visually-simple – synthesizing complex systems of determinants of financial wellbeing and financial strain to drive collaborative action.

Future work will involve engagement of decision-makers affiliated with organizations and government sectors as well as other stakeholders (concerned community members, civil society organizations) to validate the Framework in other contexts and ensure its relevance and meaningfulness for varied financial wellbeing and financial strain related issues (e.g., supporting business opportunities for Indigenous entrepreneurs). A companion guidebook with strategies and indicators has been developed to further guide design, implementation, and assessment/evaluation of initiatives related to financial wellbeing and financial strain [50, 51]. In addition to its practical applications, the Framework also constitutes a disciplinary call for coordinated multi-sectoral movement toward upstream population health approaches that will shift the distribution of financial strain and poor health outcomes. Future investigation will be carried out on how the commercial determinants of health may constrain governmental actions to address the underlying factors of financial strain and poor financial wellbeing.

## Data Availability

The datasets used and/or analysed during the current study are available from the corresponding author on reasonable request.
